# Crystal structure of *cis*-tetra­aqua­dichlorido­cobalt(II) sulfolane disolvate

**DOI:** 10.1107/S2056989014027753

**Published:** 2015-01-03

**Authors:** Mhamed Boudraa, Sofiane Bouacida, Hasna Bouchareb, Hocine Merazig, El Hossain Chtoun

**Affiliations:** a Unité de Recherche de Chimie de l’Environnement et Moléculaire Structurale, CHEMS, Université Constantine 1, 25000 , Algeria; b Département Sciences de la Matière, Faculté des Sciences Exactes et Sciences de la Nature et de la Vie, Université Oum El Bouaghi, Algeria; c Université Abdelmalek Essaadi, Faculté des Sciences, BP 2121 M’Hannech II, 93002 Tétouan, Morocco

**Keywords:** crystal structure, cobalt(II) complex, sulfolane solvate

## Abstract

In the title compound, [CoCl_2_(H_2_O)_4_]·2C_4_H_8_SO_2_, the Co^II^ cation is located on the twofold rotation axis and is coordinated by four water mol­ecules and two adjacent chloride ligands in a slightly distorted octa­hedral coordination environment. The *cisoid* angles are in the range 83.27 (5)–99.66 (2)°. The three *transoid* angles deviate significantly from the ideal linear angle. The crystal packing can be described as a linear arrangement of complex units along *c* formed by bifurcated O—H⋯Cl hydrogen bonds between two water mol­ecules from one complex unit towards one chloride ligand of the neighbouring complex. Two solvent mol­ecules per complex are attached to this infinite chain *via* O—H⋯O hydrogen bonds in which water mol­ecules act as the hydrogen-bond donor and sulfolane O atoms as the hydrogen-bond acceptor sites.

## Related literature   

For structures where the Co^II^ atom exhibits an octahedral geometry and is coordinated by water molecules, see: Waizumi *et al.* (1990 ▸); Sarangarajan *et al.* (2008 ▸). For potential applications of organic–inorganic hybrid compounds, see: Al-Ktaifani & Rukiah (2011 ▸). For related structures, see: Bouacida *et al.* (2005 ▸, 2013 ▸); Sahbani *et al.* (2014 ▸).
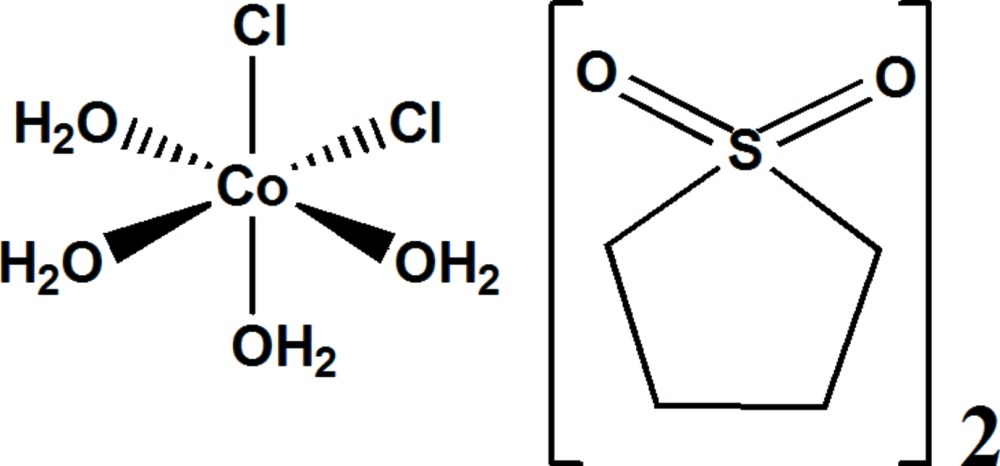


## Experimental   

### Crystal data  

[CoCl_2_(H_2_O)_4_]·2C_4_H_8_O_2_S
*M* *_r_* = 442.22
Monoclinic, 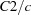

*a* = 20.062 (2) Å
*b* = 9.4284 (10) Å
*c* = 10.5882 (13) Å
β = 118.734 (5)°
*V* = 1756.2 (3) Å^3^
*Z* = 4
Mo *K*α radiation
μ = 1.55 mm^−1^
*T* = 295 K
0.21 × 0.15 × 0.09 mm

### Data collection  

Bruker APEXII diffractometer
Absorption correction: multi-scan (*SADABS*; Sheldrick, 2002 ▸) *T*_min_ = 0.649, *T*_max_ = 0.748
20238 measured reflections
5090 independent reflections
3409 reflections with *I* > 2σ(*I*)
*R*_int_ = 0.061

### Refinement  

*R*[*F* ^2^ > 2σ(*F* ^2^)] = 0.043
*wR*(*F* ^2^) = 0.129
*S* = 1.01
5090 reflections
108 parameters
4 restraints
H atoms treated by a mixture of independent and constrained refinement
Δρ_max_ = 0.90 e Å^−3^
Δρ_min_ = −1.44 e Å^−3^

### 

Data collection: *APEX2* (Bruker, 2011 ▸); cell refinement: *SAINT* (Bruker, 2011 ▸); data reduction: *SAINT*; program(s) used to solve structure: *SIR2002* (Burla *et al.*, 2005 ▸); program(s) used to refine structure: *SHELXL97* (Sheldrick, 2008 ▸); molecular graphics: *ORTEP-3 for Windows* (Farrugia, 2012 ▸) and *DIAMOND* (Brandenburg & Berndt, 2001 ▸); software used to prepare material for publication: *WinGX* (Farrugia, 2012 ▸).

## Supplementary Material

Crystal structure: contains datablock(s) I. DOI: 10.1107/S2056989014027753/im2458sup1.cif

Structure factors: contains datablock(s) I. DOI: 10.1107/S2056989014027753/im2458Isup2.hkl

**Fig. 1.**
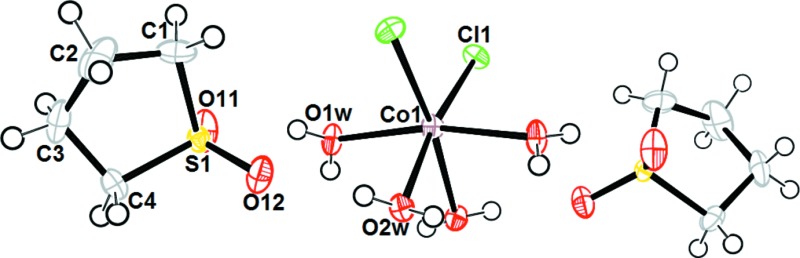
Molecular structure of (I) with displacement ellipsoids drawn at the 50% probability level. Only the asymmetric unit is labelled. H atoms are represented as small spheres of arbitrary radii.

**Fig. 2.**
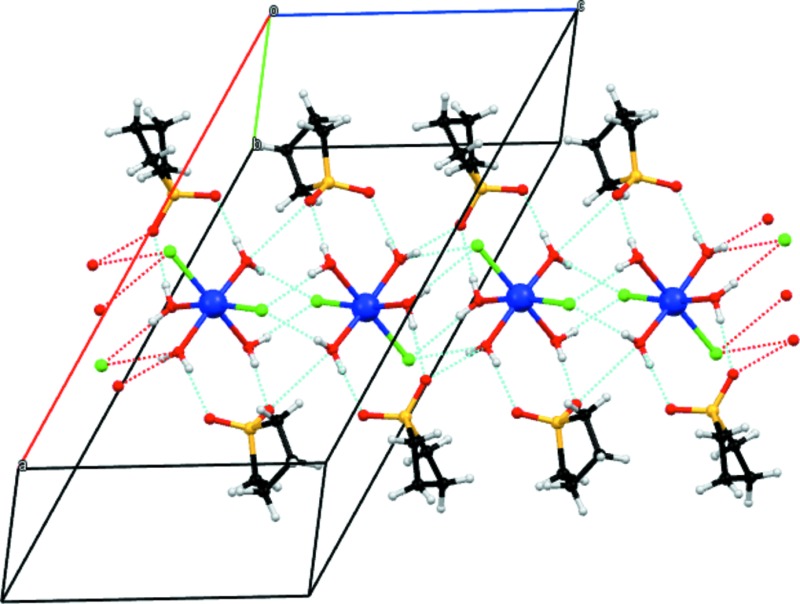
Packing diagram of (I) showing the infinite chains of complex units and solvent molecule along the c axis.

. DOI: 10.1107/S2056989014027753/im2458fig1.tif
Mol­ecular structure of (I) with displacement ellipsoids drawn at the 50% probability level. Only the asymmetric unit is labelled. H atoms are represented as small spheres of arbitrary radii.

c . DOI: 10.1107/S2056989014027753/im2458fig2.tif
Packing diagram of (I) showing the infinite chains of complex units and solvent mol­ecule along the *c* axis.

CCDC reference: 1040554

Additional supporting information:  crystallographic information; 3D view; checkCIF report

## Figures and Tables

**Table 1:** Hydrogen-bond geometry (, )

*D*H*A*	*D*H	H*A*	*D* *A*	*D*H*A*
O1*W*H1*W*Cl1^i^	0.77(3)	2.44(3)	3.1885(15)	165(3)
O1*W*H2*W*O11	0.81(2)	1.99(2)	2.782(2)	165(2)
O2*W*H3*W*Cl1^ii^	0.83(2)	2.41(2)	3.2289(16)	171(2)
O2*W*H4*W*O12	0.79(3)	2.05(3)	2.835(2)	174(3)

Symmetry codes: (i) 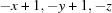; (ii) 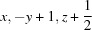.

## supplementary crystallographic information

### S1. Comment

Organic-inorganic hybrid salts have received considerable attention because of their potential applications in analytical, material and supramolecular chemistry (Al-Ktaifani *et al.*, 2011; Bouacida *et al.* 2005, 2013; Sahbani *et al.*, 2014). In this work, we report the preparation and the structural investigation of [Co(H_2_O)_4_Cl_2_] × 2 C_4_H_8_SO_2_. Since the central cobalt the Co^II^ cation is located on the twofold rotation axis the asymmetric unit of (I) consists of one one half of the complex unit and one molecule of sulfolane (Figure 1).

The structure of the compound consists of discrete tetraaquadichlorocobalt(II) complexes stacked in chains parallel to the *c* axis. The Co^II^ cation is coordinated by four water molecules and two adjacent chloride ligands in a slightly distorted octahedral geometry. The two Co—Cl distances are 2.510 (6) Å and the Co—O distances are between 2.165 (3) and 2.243 (3) Å in good agreement with that found in mineral compound CoCl_2_O_4_H_8_ (Waizumi *et al.*, 1990) and in the coordination compound [CoCl_2_(H_2_O)_4_]C_4_H_6_N_2_O_2_ (Sarangarajan *et al.*, 2008). Cisoid angles around Co atom are in the range of 83.27 (5)° to 99.66 (2)°. In the organic molecule, the S atom is tetrahedrally coordinated by two O and two C atoms. The three bonds C—C are in the range 1.516 (2)–1,531 (3) Å. In the crystal, molecules are linked by O—H···O and O—H···Cl hydrogen bonds forming chains along [001] (Figure 2). The crystal packing can be described as a linear arrangement of complex units along *c* formed by bifurcated O–H···Cl hydrogen bonds between two water molecules from one complex unit towards one chloride ligand of the neighboring complex. Two solvent molecules per complex are attached to this infinite chain via O–H···O hydrogen bonds in which water molecules act as the hydrogen bond donor and sulfolane oxygen atoms as the hydrogen bond acceptor sites.

### S2. Experimental

A solution of CoCl_2_ × 2 H_2_O (34 mg, 0.2 mmol) in water (10 ml) was added dropwise to a solution of sulfolane (24 mg, 0.2 mmol) in water (10 ml). The mixture was then refluxed with stirring for 3 h and the resulting solution was left to stand at room temperature. After several days, blue crystals were obtained and dried under vacuum (yield: 55%).

### S3. Refinement

All non-H atoms were refined with anisotropic displacement parameters. Approximate positions for all H atoms were first obtained from the difference electron density map. However, the H atoms were situated into idealized positions and the H-atoms have been refined within the riding atom approximation with C—H = 0.93 Å and *U*_iso_ = 1.2*U*_eq_(C) except for H atoms of water molecules, which were refined isotropically using the following restraints: O—H = 0.84 (2) Å, H···H = 1.45 (2) Å and *U*_iso_ = 1.5*U*_eq_(O).

### Crystal data

**Table:**

[CoCl_2_(H_2_O)_4_]·2C_4_H_8_O_2_S	*F*(000) = 916
*M**_r_* = 442.22	*D*_x_ = 1.673 Mg m^−^^3^
Monoclinic, *C*2/*c*	Mo *K*α radiation, λ = 0.71073 Å
Hall symbol: -C 2yc	Cell parameters from 5148 reflections
*a* = 20.062 (2) Å	θ = 2.5–35.1°
*b* = 9.4284 (10) Å	µ = 1.55 mm^−^^1^
*c* = 10.5882 (13) Å	*T* = 295 K
β = 118.734 (5)°	Prism, blue
*V* = 1756.2 (3) Å^3^	0.21 × 0.15 × 0.09 mm
*Z* = 4	

### Data collection

**Table:**

Bruker APEXII diffractometer	5090 independent reflections
Radiation source: sealed tube	3409 reflections with *I* > 2σ(*I*)
Graphite monochromator	*R*_int_ = 0.061
φ and ω scans	θ_max_ = 39.0°, θ_min_ = 2.3°
Absorption correction: multi-scan (*SADABS*; Sheldrick, 2002)	*h* = −34→35
*T*_min_ = 0.649, *T*_max_ = 0.748	*k* = −16→16
20238 measured reflections	*l* = −18→14

### Refinement

**Table:**

Refinement on *F*^2^	Primary atom site location: structure-invariant direct methods
Least-squares matrix: full	Secondary atom site location: difference Fourier map
*R*[*F*^2^ > 2σ(*F*^2^)] = 0.043	Hydrogen site location: inferred from neighbouring sites
*wR*(*F*^2^) = 0.129	H atoms treated by a mixture of independent and constrained refinement
*S* = 1.01	*w* = 1/[σ^2^(*F*_o_^2^) + (0.0666*P*)^2^] where *P* = (*F*_o_^2^ + 2*F*_c_^2^)/3
5090 reflections	(Δ/σ)_max_ = 0.007
108 parameters	Δρ_max_ = 0.90 e Å^−^^3^
4 restraints	Δρ_min_ = −1.44 e Å^−^^3^

### Special details

**Table:**

Geometry. All e.s.d.'s (except the e.s.d. in the dihedral angle between two l.s. planes) are estimated using the full covariance matrix. The cell e.s.d.'s are taken into account individually in the estimation of e.s.d.'s in distances, angles and torsion angles; correlations between e.s.d.'s in cell parameters are only used when they are defined by crystal symmetry. An approximate (isotropic) treatment of cell e.s.d.'s is used for estimating e.s.d.'s involving l.s. planes.
Refinement. Refinement of *F*^2^ against ALL reflections. The weighted *R*-factor *wR* and goodness of fit *S* are based on *F*^2^, conventional *R*-factors *R* are based on *F*, with *F* set to zero for negative *F*^2^. The threshold expression of *F*^2^ > σ(*F*^2^) is used only for calculating *R*-factors(gt) *etc*. and is not relevant to the choice of reflections for refinement. *R*-factors based on *F*^2^ are statistically about twice as large as those based on *F*, and *R*- factors based on ALL data will be even larger.

### Fractional atomic coordinates and isotropic or equivalent isotropic displacement parameters (Å^2^)

**Table:**

	*x*	*y*	*z*	*U*_iso_*/*U*_eq_	
Co1	0.5	0.47467 (3)	0.25	0.01473 (8)	
S1	0.76287 (2)	0.53220 (4)	0.58938 (4)	0.01334 (9)	
Cl1	0.43290 (2)	0.30294 (4)	0.04608 (4)	0.01647 (9)	
O1W	0.59916 (7)	0.49821 (15)	0.22088 (14)	0.0177 (2)	
H1W	0.5953 (15)	0.556 (2)	0.168 (2)	0.027*	
H2W	0.6412 (11)	0.483 (3)	0.288 (2)	0.027*	
O2W	0.54617 (7)	0.65037 (13)	0.41295 (13)	0.0155 (2)	
H3W	0.5200 (12)	0.669 (3)	0.452 (2)	0.023*	
H4W	0.5887 (12)	0.631 (3)	0.467 (3)	0.023*	
C3	0.90875 (11)	0.5145 (2)	0.7520 (3)	0.0298 (5)	
H3A	0.955	0.5538	0.8296	0.036*	
H3B	0.9206	0.4724	0.6815	0.036*	
C4	0.84886 (10)	0.6288 (2)	0.6830 (2)	0.0240 (4)	
H4A	0.8584	0.686	0.6172	0.029*	
H4B	0.8475	0.69	0.7554	0.029*	
C1	0.79391 (13)	0.3742 (2)	0.6942 (2)	0.0293 (4)	
H1A	0.7618	0.3529	0.737	0.035*	
H1B	0.7924	0.2945	0.635	0.035*	
C2	0.87487 (14)	0.4035 (3)	0.8102 (3)	0.0386 (6)	
H2A	0.9047	0.317	0.8342	0.046*	
H2B	0.8752	0.4386	0.8966	0.046*	
O12	0.70254 (8)	0.60180 (16)	0.60336 (16)	0.0245 (3)	
O11	0.74827 (8)	0.50189 (17)	0.44424 (16)	0.0273 (3)	

### Atomic displacement parameters (Å^2^)

**Table:**

	*U*^11^	*U*^22^	*U*^33^	*U*^12^	*U*^13^	*U*^23^
Co1	0.01483 (14)	0.01815 (15)	0.01223 (15)	0	0.00731 (12)	0
S1	0.01081 (15)	0.01611 (17)	0.01057 (17)	−0.00112 (12)	0.00313 (13)	0.00104 (12)
Cl1	0.02063 (18)	0.01729 (17)	0.01137 (16)	−0.00469 (13)	0.00760 (14)	−0.00251 (13)
O1W	0.0121 (5)	0.0302 (7)	0.0117 (5)	0.0022 (4)	0.0064 (4)	0.0051 (5)
O2W	0.0149 (5)	0.0186 (5)	0.0130 (5)	−0.0020 (4)	0.0067 (4)	−0.0022 (4)
C3	0.0136 (7)	0.0444 (12)	0.0283 (11)	0.0051 (7)	0.0074 (7)	−0.0038 (9)
C4	0.0173 (7)	0.0219 (8)	0.0277 (9)	−0.0067 (6)	0.0068 (7)	−0.0036 (7)
C1	0.0397 (12)	0.0158 (8)	0.0332 (11)	0.0009 (7)	0.0182 (10)	0.0080 (7)
C2	0.0326 (11)	0.0517 (14)	0.0278 (11)	0.0189 (10)	0.0115 (9)	0.0219 (10)
O12	0.0160 (6)	0.0326 (7)	0.0230 (7)	0.0034 (5)	0.0079 (5)	−0.0044 (5)
O11	0.0188 (6)	0.0506 (9)	0.0112 (6)	−0.0003 (6)	0.0060 (5)	−0.0021 (6)

### Geometric parameters (Å, º)

**Table:**

Co1—O1W	2.1660 (13)	O2W—H4W	0.79 (2)
Co1—O1W^i^	2.1660 (13)	C3—C4	1.515 (3)
Co1—O2W	2.2455 (12)	C3—C2	1.529 (4)
Co1—O2W^i^	2.2455 (12)	C3—H3A	0.97
Co1—Cl1	2.5102 (5)	C3—H3B	0.97
Co1—Cl1^i^	2.5102 (5)	C4—H4A	0.97
S1—O12	1.4456 (14)	C4—H4B	0.97
S1—O11	1.4478 (16)	C1—C2	1.518 (3)
S1—C4	1.7730 (18)	C1—H1A	0.97
S1—C1	1.7820 (19)	C1—H1B	0.97
O1W—H1W	0.758 (16)	C2—H2A	0.97
O1W—H2W	0.812 (17)	C2—H2B	0.97
O2W—H3W	0.830 (16)		
			
O1W—Co1—O1W^i^	168.24 (8)	H3W—O2W—H4W	115 (2)
O1W—Co1—O2W	88.05 (5)	C4—C3—C2	106.16 (18)
O1W^i^—Co1—O2W	83.27 (5)	C4—C3—H3A	110.5
O1W—Co1—O2W^i^	83.27 (5)	C2—C3—H3A	110.5
O1W^i^—Co1—O2W^i^	88.05 (5)	C4—C3—H3B	110.5
O2W—Co1—O2W^i^	84.92 (7)	C2—C3—H3B	110.5
O1W—Co1—Cl1	95.34 (4)	H3A—C3—H3B	108.7
O1W^i^—Co1—Cl1	92.24 (4)	C3—C4—S1	103.73 (13)
O2W—Co1—Cl1	171.61 (4)	C3—C4—H4A	111
O2W^i^—Co1—Cl1	87.85 (3)	S1—C4—H4A	111
O1W—Co1—Cl1^i^	92.24 (4)	C3—C4—H4B	111
O1W^i^—Co1—Cl1^i^	95.34 (4)	S1—C4—H4B	111
O2W—Co1—Cl1^i^	87.85 (3)	H4A—C4—H4B	109
O2W^i^—Co1—Cl1^i^	171.61 (4)	C2—C1—S1	105.63 (15)
Cl1—Co1—Cl1^i^	99.66 (2)	C2—C1—H1A	110.6
O12—S1—O11	116.50 (9)	S1—C1—H1A	110.6
O12—S1—C4	110.32 (9)	C2—C1—H1B	110.6
O11—S1—C4	109.92 (10)	S1—C1—H1B	110.6
O12—S1—C1	112.01 (10)	H1A—C1—H1B	108.7
O11—S1—C1	109.14 (10)	C1—C2—C3	107.97 (17)
C4—S1—C1	97.26 (10)	C1—C2—H2A	110.1
Co1—O1W—H1W	114 (2)	C3—C2—H2A	110.1
Co1—O1W—H2W	120 (2)	C1—C2—H2B	110.1
H1W—O1W—H2W	118 (3)	C3—C2—H2B	110.1
Co1—O2W—H3W	114.7 (17)	H2A—C2—H2B	108.4
Co1—O2W—H4W	106.8 (18)		
			
C2—C3—C4—S1	−42.0 (2)	O11—S1—C1—C2	115.94 (17)
O12—S1—C4—C3	140.20 (15)	C4—S1—C1—C2	1.88 (19)
O11—S1—C4—C3	−89.99 (17)	S1—C1—C2—C3	−27.0 (2)
C1—S1—C4—C3	23.45 (17)	C4—C3—C2—C1	45.6 (3)
O12—S1—C1—C2	−113.53 (17)		

Symmetry code: (i) −*x*+1, *y*, −*z*+1/2.

### Hydrogen-bond geometry (Å, º)

**Table:**

*D*—H···*A*	*D*—H	H···*A*	*D*···*A*	*D*—H···*A*
O1*W*—H1*W*···Cl1^ii^	0.77 (3)	2.44 (3)	3.1885 (15)	165 (3)
O1*W*—H2*W*···O11	0.81 (2)	1.99 (2)	2.782 (2)	165 (2)
O2*W*—H3*W*···Cl1^iii^	0.83 (2)	2.41 (2)	3.2289 (16)	171 (2)
O2*W*—H4*W*···O12	0.79 (3)	2.05 (3)	2.835 (2)	174 (3)

Symmetry codes: (ii) −*x*+1, −*y*+1, −*z*; (iii) *x*, −*y*+1, *z*+1/2.
