# Supplementary figures and images for: Crystal structure of cis-tetra­aqua­dichlorido­cobalt(II) sulfolane disolvate
Source: Acta Crystallogr E Crystallogr Commun. 2015 Jan 3;71(Pt 2):m16–7. doi: 10.1107/S2056989014027753 (PMC4384590; doi:10.1107/S2056989014027753)

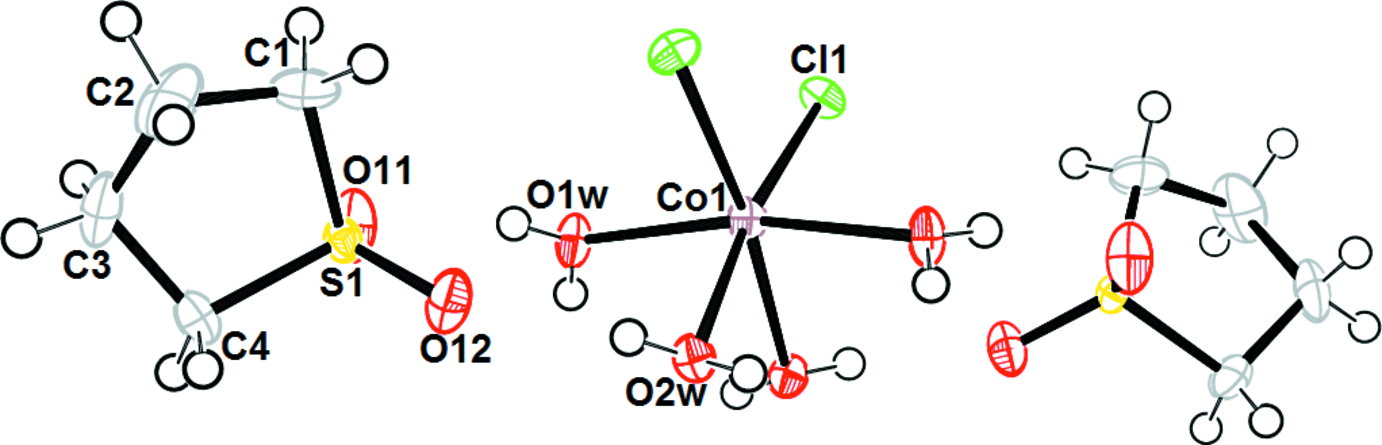

Supplement: Supplementary file 3 [file e-71-00m16-fig1.tif]

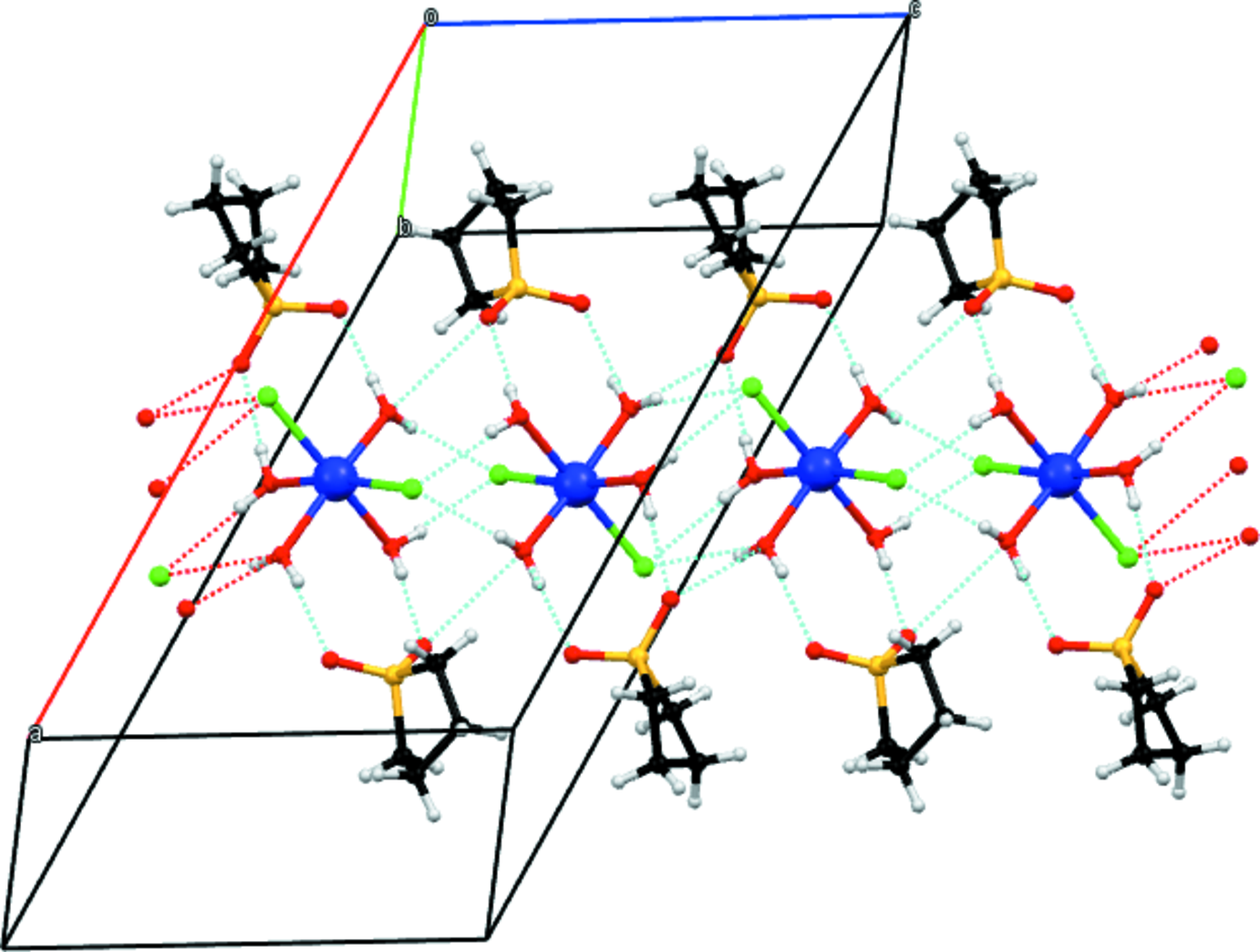

Supplement: Supplementary file 4 [file e-71-00m16-fig2.tif]
